# Stem Cell Enriched Fat Grafts versus Autologous Fat Grafts in Reconstructive Surgery: Systematic Review and Meta-Analysis

**DOI:** 10.1007/s00266-023-03421-z

**Published:** 2023-06-21

**Authors:** Mohammad Karam, Ahmad Abul, Shafiq Rahman

**Affiliations:** 1 Department of Opthalmology, McGill University, Montreal, Canada; 2 Division of Surgical and Interventional Science, University College London, Gower Street, London, WC1E6BT United Kingdom; 3 Department of Plastic Surgery, Leeds Teaching Hospitals, Leeds General Infirmary, Leeds, United Kingdom

**Keywords:** Stem cell-enrichment fat grafts, Volume retention, Patient satisfaction

## Abstract

**Objective:**

To compare the outcomes of stem cell-enrichment fat grafting (SCEFG) versus autologous fat grafting (AFG) for reconstructive purposes.

**Methods:**

A systematic review and meta-analysis was performed as per the preferred reporting items for systematic reviews and meta-analyses. Guidelines and a search of electronic information was conducted to identify all Randomised Controlled Trials (RCTs), case-control studies and cohort studies comparing the outcomes of SCEFG versus AFG. Volume retention, fat necrosis, cancer recurrence, redness and swelling, infection, and cysts were primary outcome measures. Secondary outcome measures included patient satisfaction post-surgery, scar assessment, operation time and number of fat grafting sessions. Fixed and random effects modelling were used for the analysis.

**Results:**

16 studies enrolling 686 subjects were selected. Significant differences between the SCEFG and AFG groups were seen in mean volume retention (standardised mean difference = 3.00, *P* < 0.0001) and the incidence of redness and swelling (Odds Ratio [OR] = 441,* P* = 0.003). No significant difference between the two groups in terms of fat necrosis (OR = 2.23,* P* = 0.26), cancer recurrence (OR = 1.39,* P* = 0.58), infection (OR = 0.30,* P* = 0.48) and cysts (OR = 0.88,* P* = 0.91). For secondary outcomes, both cohorts had similar results in patient satisfaction, scar assessment and number of fat grafting sessions. Operation time was longer for the intervention group.

**Conclusions:**

SCEFG offers better outcomes when compared to AFG for reconstructive surgery as it improves the mean volume retention and does not worsen patient satisfaction and surgical complications except for self-limiting redness and swelling. Further clinical trials are recommended to support this argument and validate the use of SCEFG in clinical practice.

**Level of Evidence III:**

This journal requires that authors assign a level of evidence to each article. For a full description of these Evidence-Based Medicine ratings, please refer to the Table of Contents or the online Instructions to Authors www.springer.com/00266.

## Introduction

Over the last two decades, techniques of fat transplantation have significantly improved from simple free transfers of intact adipose tissue to free composite fat-cell transplantation techniques [1]. There are various indications of fat grafting, ranging from breast and craniofacial reconstruction to improvement of joint mobility after surgery for ankylosis [2].

The application of autologous fat grafts (AFG) for soft tissue augmentation has increased in popularity, creating an inexpensive and readily available product for lipo-grafting [3]. Krastev et al. (2018) conducted a systematic review that demonstrated the effectiveness of AFGs in treating soft-tissue abnormalities with minimal complications [4]. Conversely, other studies mention that there is a lack of reliable scientific evidence on the long-term viability of the transferred fat [3]. Traditional techniques of AFG for lipo-sculpting have recorded poor rates of volume retention ranging from 25 to 80%, which often requires additional procedures to optimise cosmetic outcomes [5–8].

Stem cells have been able to augment tissue regeneration within a host matrix by differentiating along specific cell lineages [9]. This principle has been utilised in lipofilling when reconstructing defects through the enrichment of fat grafts with stromal vascular fraction [10]. Stem cell enriched fat grafts (SCEFG) have been routinely adapted to enrich harvested fat with adipose-derived stromal cells which can increase graft take and volume retention by differentiation into mature adipocytes [11, 12]. Surgeons have been able to correct deformities in the breast, face as well as other anatomical regions utilising this method [13–17].

Although randomised controlled trials (RCTs) and non-randomised studies have shown promising results [17–32], no study in the literature currently analyses the outcomes of all comparative studies. This is the first systematic review and meta-analysis focusing on all comparative studies assessing SCEFG versus routine AFG for soft tissue reconstruction.

## Methods

This systematic review and meta-analysis were conducted according to the Preferred Reporting Items for Systematic Reviews and Meta-analyses (PRISMA) statement standards [33], but it was not registered at the International Prospective Register of Systematic Reviews.

### Eligibility Criteria

All comparative studies including randomised as well as non-randomised controlled trials and observational studies comparing SCEFG versus routine AFG for reconstructive purposes were included. SCEFG was the intervention of interest and AFGs was the comparator. All patients were included irrespective of age, gender, co-morbidity status or anatomical region reconstructed as long as they belonged to either a study or control group. Case reports and cohort studies where no comparison was conducted were excluded from the review process.

### Outcome Measures

The primary outcome measures included volume retention, fat necrosis, cancer recurrence, redness and swelling, infection, and cysts. Secondary outcomes included patient satisfaction post-surgery, scar assessment, operation time and number of fat grafting sessions.

### Literature Search Strategy

Two authors independently searched the electronic databases of MEDLINE, EMBASE, CINAHL and the Cochrane Central Register of Controlled Trials (CENTRAL). The last search was conducted on 1^st^ August 2022. The search strategy was adapted according to the thesaurus headings, search operators and limits in each of the above databases. The search terms for our intervention of interest consisted of “stem cell enriched”, “stromal vascular fraction enriched”, “ADSC”, “ASCs”, “ADRCs”, “progenitor enriched”, “call assisted lipotransfer”. Search terminologies used for the control group consisted of “fat graft”, “fat harvest”, “fat transplantation”, “autologous fat harvest”, “lipofilling”, “fat transfer”, “lipograft”, All terms were combined with adjuncts of “and” as well as “or”. To extend the screening for eligible articles, the bibliographic lists were also reviewed for the relevant studies. No search restriction was placed on language as a limiting factor.

### Selection of Studies

The titles and abstracts of articles retrieved from the literature were independently assessed by two authors. Articles that met the eligibility criteria were selected after their full texts were reviewed. A consultation was obtained from an independent third author for any discrepancies in study selection.

### Data Extraction and Management

A Microsoft Excel data extraction spreadsheet was amalgamated that abided with Cochrane’s data collection form for intervention reviews. A pilot test was conducted with the spreadsheet extracting data from random articles and adapting it as needed. Three authors independently extracted and recorded data.

### Data Synthesis

The authors aimed to perform a meta-analysis for outcomes reported by at least three studies. The odds ratio (OR) was reserved for dichotomous variables whereas the mean difference (MD) was aimed to assess continuous variables for the study and control groups.

Review Manager 5.3 and Microsoft excel was used for data analysis and the fixed and random effects models were used. Reported outcomes were given in forest plots at 95% confidence intervals (CIs).

Heterogeneity was assessed using the Cochran Q test (χ^2^) and we aimed to quantify inconsistency by calculating I^2^ which was interpreted per the following: 0% to 25% (low heterogeneity); 25% to 75% (moderate heterogeneity); and 75% to 100% (considerable heterogeneity). The authors also intended to develop funnel plots and asses their symmetry to review publication bias, however, only if a minimum of 10 studies were available.

### Sensitivity and Sub-Group Analyses

To identify areas of heterogeneity and assess the robustness of results, the authors aimed to perform additional analyses for outcomes that were reported by at least four studies.

### Methodological Quality and Risk Of Bias Assessment

Two authors independently assessed the methodological quality as well as the risk of bias for articles matching the inclusion criteria. Cochrane’s tool for evaluating the risk of bias for randomised trials was used. Domains assessed included selection bias, performance bias, detection bias, attrition bias, reporting bias, and other sources. It classifies studies into low, unclear, and high risk of bias. In addition, Newcastle-Ottawa Scale [34] was used for the assessment of bias of non-randomised studies in terms of three domains: selection, comparability and exposure. It uses a star scoring system with a maximum total score of nine stars for each study.

## Results

### Literature Search Results

A literature search reported 248 articles in total which were reviewed by two independent authors to filter out duplicates, abstracts, review articles, studies without the intervention of interest as well as those without comparative control groups and reports involving non-human subjects. Sixteen studies were selected which met the eligibility criteria of which included 5 randomized control trials and 11 non-randomised case-control studies.

### Description of Studies

**Fig. 1:**
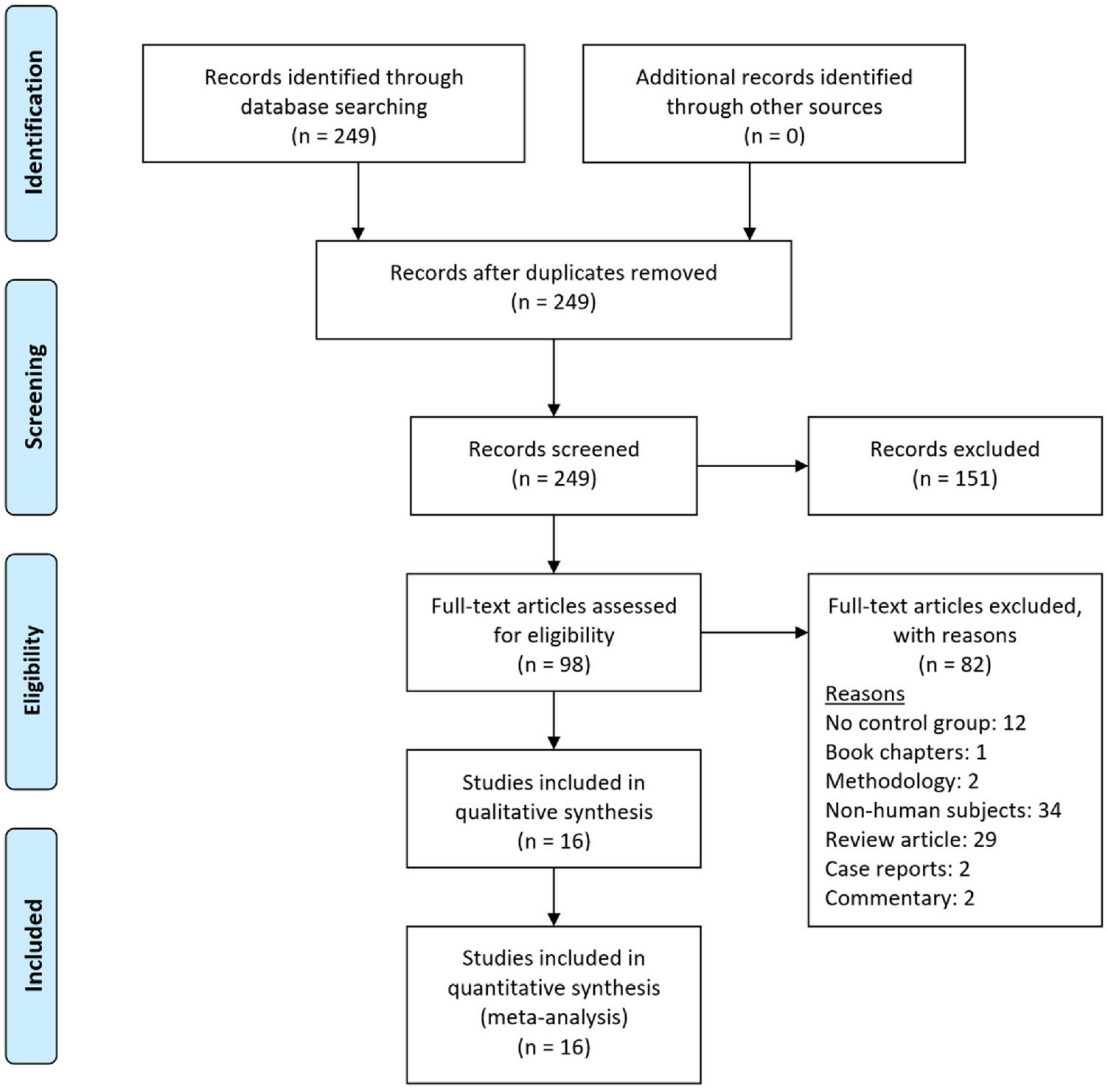
Prisma Flow Diagram. the PRISMA diagram details the search and selection processes applied during the overview. prisma, preferred reporting items for systematic reviews and meta-analyses

Table 1 summarizes the baseline characteristic of the included studies. The studies were homogenous in the study population and interventions of interest comparing SCEFG and AFG in reconstructive surgery. The current meta-analysis included a total of 16 studies with a total sample size of 686 grafts. Eight studies focused on facial reconstruction, six studies on breast reconstruction and two studies on limb reconstruction (Fig. 1).

**Table 1:** Baseline characteristics of the included studies

Study (year)	Journal, Country	Study design	Age	Sex (male:female)	Total sample (intervention: control)	Anatomical location	Interventions compared	Method of stem cell enrichment	Volume retention assessment
Yoshimura et al. (2008)	Dermatologic surgery, Japan	Case control study	37.5 years (mean)	2:4	6 (3:3)	Face	CAL versus conventional lipoinjection (non-CAL)	The liposuction aspirate was digested with 0.075% collagenase for 30 minutes at 37 °C, which separated mature adipocytes and connective tissue from the stromal vascular fraction (SVF) containing ASCs. The SVF was then centrifuged and rinsed to isolate the cells, which took about 90 minutes.	High resolution digital camera (Model D30, Canon)
Sterodimas et al. (2011)	Aesthetic surgery journal, Brazil	RCT	45.15 years (mean)	5:15	20 (10:10)	Face	ADSC enriched lipografts versus autologous fat transplant	The SVF was isolated from the aspirated fat through a process involving collagenase digestion, centrifugation, and separation. The SVF and purified fat were mixed and transferred to syringes for injection	NR
Gentile et al. (2012)	Stem Cells translational medicine, Italy	Cohort Study	19–60 years (range)	0:20	20 (10:10)	Abdominal to breast	SVF enhanced autologous fat graft versus centrifuged fat grafting injection	Half of lipoaspirate placed in Celution system, Blood free lipid removed by wash cycle. Celase835 enzymatically digested tissue releasing SVF. Centrifugation -> extraction of SVF Added to 2^nd^ half of lipoaspirate that is washed and filtrated.	MRI
Koh et al. (2012)	Annals plastic surgery, South Korea	RCT	28 years (mean)	5:5	10 (5:5)	Abdominal to face	Microfat grafts and simultaneous human ASC injection versus microfat grafts alone	Human ASCs were extracted from adipose tissue and isolated using standard protocols from the International Society for Cellular Therapy [46].	3-dimentional CT scan
Li et al. (2012)	Dermatologic surgery, China	Case control study	Intervention: 29.5 years (mean) Control: 29.1 years (mean)	0:38	38 (26:12)	Abdominal or thigh to face	Transplantation with fat mixed with SVF versus fat grafting alone	The suctioned fat was digested with collagenase and then processed to obtain SVF, by separating mature adipocytes, resuspending in erythrocyte lysis buffer, washing to remove collagenase, and collecting the harvested pellet.	CT
Chang et al. (2013)	Dermatologic surgery, China	Case control study	27.5 years (mean)	8:12	20 (10:10)	Abdominal to face	SVF supplemented fat grafting versus fat grafting alone	The SVF was isolated from the fatty tissue using a modification of a method described by Zuk and colleagues. The fluid was filtered, washed and centrifuged to obtain the SVF, which was then mixed with the harvested adipose tissue and injected into subcutaneous tissue in a sterile operating room.	CT
Kølle et al. (2013)	Lancet, Denmark	RCT	28.4 years (mean)	NR	20 (10:10)	Abdominal to arm	ASC-enriched fat graft versus non-enriched fat graft	ASCs were isolated and expanded in a certified laboratory using GMP-grade reagents. They were then cultured for 2 weeks with a mixture of Dulbecco’s modified Eagle’s medium, antibiotics, heparin, and human platelet lysate, with regular media changes and checks for contamination.	MRI
Peltoniemi et al. (2013)	Journal of plastic, reconstructive & aesthetic surgery (jpras), Finland	Case control study	Intervention: 51 years (mean); 29–58 years (range) control: 39 years (mean); 33–63 years (range)	0:18	18 (10:8)	Breast	WAL enriched with stromal stem cells versus WAL alone	The first part of the harvested fat using the WAL technique is processed in the Celution System. The ASCs are isolated and separated from other cells through enzymatic digestion and multiple cycles of automatic centrifugation and washing. The final ASC-rich stroma is mixed with the ordinary fat graft and immediately injected	MRI
Tanikawa et al. (2013)	Plastic and reconstructive surgery journal, Brazil	RCT	Intervention: 18.7 ± 7.6 years (mean ± SD) control: 12.1 ± 2.2 years (mean ± SD)	5:9	14 (7:7)	Lower abdominal to face	Fat grafting with supplementation of ADSC stromal cells versus fat grafting without supplementation of ADSC	The liposuction aspirates were digested with 0.15% collagenase type IA to separate mature adipocytes and connective tissue from the SVF which contains the adipose-derived stromal cells. The SVF was resuspended in water and added to the aspirated fat, then transferred into syringes for injection after mixing and waiting for cell adherence.	CT
Bashir et al. (2019)	Stem Cells international, Pakistan	Quasi-experimental Study	Intervention: 30 ± 11 years (mean ± SD) control: 21 ± 5 years (mean ± SD)	11:26	37 (16:21)	Abdominal or lateral thigh to face	Stem cells mixed with fat graft versus fat graft only	Fat harvest for isolation and expansion of ASCs -> expansion of ASCs -> fat harvest, preparation, and enrichment with ASCs (2–4 weeks) -> pre-enriched fat transplant	Ultrasonography
Gentile et al. (2015)	Plastic and reconstructive surgery — global open (prs global open), Italy	Case control study	19–60 (range)	0:50	50 (40:10)	Breast	SVF enhanced fat grafts versus centrifuged fat alone	Half of lipoaspirate placed in Celution system, Blood free lipid removed by wash cycle. Celase835 enzymatically digested tissue releasing SVF. Centrifugation using Coleman procedure -> extraction of SVF added to 2^nd^ half of lipoaspirate that is also washed and filtrated.	MRI
Sasaki (2015)	Aesthetic plastic surgery, USA	Case control study	Intervention: 63.3 years (mean) control: 60.5 years (mean)	4:126	130 (38:92)	Abdominal or hip rolls to face	SVF- assisted fat grafting versus combination of PRP/SVF- assisted fat grafting versus conventional fat grafting	A portion of processed adipose tissue is loaded into the Celution System for automated SVF isolation. The SVF is extracted through a process of collagenase digestion, washing, and centrifugation, resulting in a SVF-filled syringe that is mixed with processed fat within 2–4 hours.	3D Vectra imaging
Tissiani et al. (2016)	Stem cells international, Brazil	Case control study	49.68 years (mean)	0:17	17 (9:8)	Breast	Fat grafts with SVF enrichment versus Fat grafts without SVF enrichment	The SG group collected 600cc of fat which was centrifuged in 50cc conic tubes. The fat was then digested with collagenase and mixed with the SVF for 15 minutes for cell adherence to occur.	MRI
Calabrese et al. (2018)	European review for medical and pharmacological sciences, Italy	Cohort study	Intervention: 48.8 years (mean) control: 50.3 years (mean)	0:105	105 (41:64)	Breast	SVF enriched adipose tissue grafting versus AFG	Aspirated adipose tissue divided into 2 parts. 1^st^ part added to Celution system, ADRCs released from bound matrix and concentrated. 2^nd^ part was purified by gravity sedimentation. 1^st^ part added to 2^nd^ part creating ADRC enriched fat graft.	NR
Gentile et al. (2019)	Journal of clinical medicine, Italy	Case control study	56.24 ± 11.44 years (mean ± SD); 25–85 years (range)	171:0	171 (121:50)	Breast	EF-e-A versus engineered fat graft that was not enhanced with ASCs	Half of lipoaspirate placed in Celution system, Blood free lipid removed by wash cycle. Celase835 enzymatically digested tissue releasing SVF. Centrifugation using Coleman procedure -> extraction of SVF added to 2^nd^ half of lipoaspirate that is also washed and filtrated.	MRI
Malik et al. (2019)	Indian journal of orthopaedics, India	RCT	32.10 years (mean); 22–45 years (range)	NR	10 (5:5)	Amputation stump	AFG enriched with SVF versus autologous fat graft alone	The lipoaspirate was processed to isolate the SVF and purified fat. The SVF was treated with 0.075% collagenase, separated through centrifugation, and analyzed for viability. The purified fat was separated from the rest of the lipoaspirate solution and both the SVF and fat were combined for injection	MRI

NR: not reported; SVF: stromal vascular fraction; ASC: adipose-derived stem cells; CAL: cell-assisted lipotransfer; EF-e-A: engineered fat graft enhanced with adipose-derived stromal vascular fraction cells; WAL: water-assisted lipotransfer; PRP: platelet‐rich plasm ADRC: Adipose derived regenerative cells; CT: Computed tomography scan; MRI: Magnetic resonance imaging. 3D: 3 dimentional

### Primary Outcomes

#### Volume Retention

**Fig. 2:**
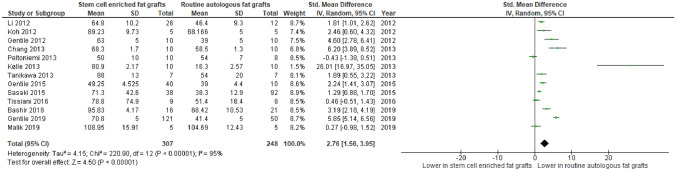
Forest plot for mean difference of cal versus aft - Volume retention. Quantitative analysis showing a statistically higher mean percentage volume retention in stem cell enhanced lipo-transfer compared with routine aft

In Fig. 2, mean volume retention was reported in 13 studies enrolling 555 subjects. There was a statistically significant difference seen in the mean difference analyses showing a higher mean percentage volume retention for the SCEFG group than the AFG group (Standardised MD = 2.76, CI = 1.56 to 3.95,* P* < 0.000001). A considerable level of heterogeneity was found amongst the studies (I^2^ = 95%,* P* < 0.00001).

Moreover, Yoshimura et al. revealed that the volume of fat retention showed a minimal change in the SCEFG group whereas it continued to be reduced in the AFG group. In addition, Malik et al. reported that there’s a more significant increase in the mean fat area on MRI scans pre-operatively and 6 months post-operatively in the SCEFG (17.8800 to 26.8280;* P* = 0.009) than the control group (24.0720 to 28.7620;* P* = 0.158).

#### Fat Necrosis

**Fig. 3:**
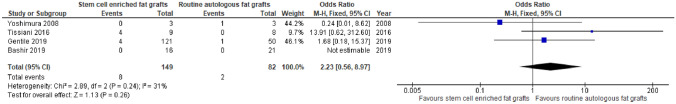
Forest plot for odds ratio of stem cell enriched fat versus routine autologous fat transfer – fat necrosis. Quantitative analysis showing a statistically insignificant lower rate of fat necrosis in stem cell enhanced lipo-transfer compared with routine autologous fat transfer

In Fig. 3, the rate of fat necrosis was reported in three studies enrolling 194 subjects. There was a statistically insignificant difference seen in the odds ratio analyses showing a lower rate of fat necrosis for the SCEFG group than the routine group (OR = 2.23, CI = 0.56 to 8.97,* P* = 0.26). A moderate level of heterogeneity was found amongst the studies (I^2^ = 31%,* P* = 0.24). Furthermore, Kølle et al reported a lower mean area of necrosis in the SCEFG group than the control group, with values of 4.6% and 16.1% respectively. Gentile et al. (2015), however, reported no complications in any patient in both groups.

#### Cancer Recurrence

**Fig. 4:**
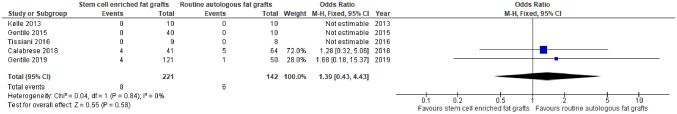
Forest plot for odds ratio of stem cell enriched fat versus routine autologous fat transfer – cancer recurrence. Quantitative analysis showing a statistically insignificant higher rate of cancer recurrence in stem cell enhanced lipo-transfer compared with routine autologous fat transfer

In Fig. 4, the rate of cancer recurrence was reported in five studies enrolling 363 subjects. There was a statistically insignificant difference seen in the odds ratio analyses showing a higher rate of cancer recurrence for the SCEFG group than the AFG group (OR = 1.39, CI = 0.43 to 4.43,* P* = 0.58). A low level of heterogeneity was found amongst the studies (I^2^ = 0%,* P* = 0.84).

#### Redness and Swelling

**Fig. 5:**
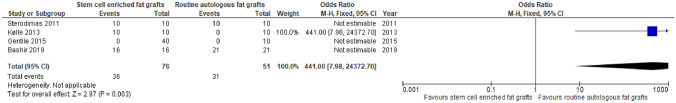
Forest plot for odds ratio of stem cell enriched fat versus routine autologous fat transfer – redness and swelling. Quantitative analysis showing a statistically significant higher rate of redness and swelling in stem cell enhanced lipo-transfer compared with routine autologous fat transfer

In Fig. 5, the rate of redness and swelling was reported in three studies enrolling 90 subjects. There was a statistically significant difference seen in the odds ratio analyses showing a higher rate of redness and swelling for the SCEFG group than the AFG group (OR = 441, CI = 7.98 to 24372.70,* P* = 0.003). Additionally, Chang et al. reported improvement in colour at the site in both groups. Li et al., Tanikawa et al. and Bashir et al. all reported swelling in both groups in the early postoperative period that was self-limiting with no further complications. Bashir et al. also reported bruising in 11 (69%) patients in SCEFG enriched group, whereby bruising occurred after 36 sessions and resolved in 2–3 weeks. Similarly, Sasaki described transient swelling experienced by all patients lasting for 2–3 weeks until full recovery with no difference between both groups, whereby there were no recorded incidences of hematomas or tissue loss.

#### Infection

**Fig. 6:**
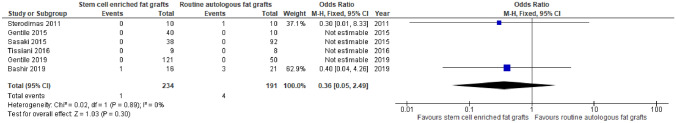
Forest plot for odds ratio of stem cell enriched fat versus routine autologous fat transfer – infection. Quantitative analysis showing a statistically insignificant lower rate of infection in stem cell enhanced lipo-transfer compared with routine autologous fat transfer

In Fig. 6, the rate of infection was reported in six studies enrolling 425 subjects. There was no significant difference seen in the odds ratio analysis between the SCEFG group and the AFG group (OR = 0.36, CI = 0.05 to 2.49,* P* = 0.30). Heterogeneity was revealed to be low between the studies (I^2^= 0%, *P*=0.89). Moreover, Kølle et al. and Tanikawa et al. did not report any adverse events or surgical complications respectively.

#### Cysts

**Fig. 7:**
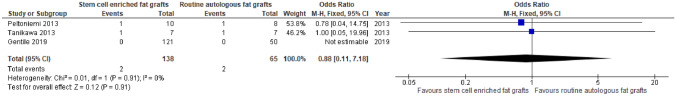
Forest plot for odds ratio of stem cell enriched fat versus routine autologous fat transfer – cysts. Quantitative analysis showing a statistically insignificant similar rate of cysts in stem cell enhanced lipo-transfer compared with routine autologous fat transfer

In Fig. 7, the rate of cysts was reported in three studies enrolling 203 subjects. There was no statistically significant difference seen in the odds ratio analyses showing a similar rate of cysts for the SCEFG group and the routine group (OR = 0.88, CI = 0.11 to 7.18,* P* = 0.91). A low level of heterogeneity was found amongst the studies (I^2^ = 0%,* P* = 0.91). In addition, Gentile et al. (2015) highlighted oily cysts detected by ultrasound in 45.83% of study group at 12 months after the last lipofilling.

#### Patient Satisfaction Post-Surgery

Sterodimas et al. highlighted more satisfaction in the SCEFG group than the non-enriched group at 6 months; however, no difference was noted at 18 months. Visual analog scale was used by Koh et al. for the assessment of patient satisfaction, highlighting a higher score for the intervention group (4.5) than the control group (3.1). Similarly, Bashir et al. did not only report a significantly higher patient satisfaction score (mean score: 2.52 ± 0.521 [ADSC enriched group] vs. 4.25 ± 0.68 [routine AFT group]) in the intervention group but also a higher physician satisfaction score (mean score: 3.69 ± 0.79 [ADSC enriched group] vs. 2.14 ± 0.36 [routine AFT group]).

Gentile et al. (2012) reported satisfaction from all the patients in both groups in terms of the resulting softness, texture and contour. In addition, Gentile et al. (2015) reported satisfaction with the same characteristics from all the patients in the study group, although there was no report of the satisfaction of the control group. Similarly, Sasaki did not compare the satisfaction of both groups; however, 90–95% of patients were satisfied.

No significant differences between the intervention and control groups in patient satisfaction with the results of fat grafting were reported by Tissiani et al. and Gentile et al. (2019), with* P* values of 0.52 and 0.603 in turn.

#### Scar Assessment

Clinical assessment of scars by Malik et al. was performed using the observer scar assessment scale (POSAS) score preoperatively and at 1 and 6 months postoperatively, whereby both groups showed similar improvement in scar characteristics. Li et al. reported that no scars were present in patients during follow‐up.

#### Operative Time

Both Peltoniemi et al. and Tanikawa et al. reported that the operation time was longer in the stem-cell enriched group than the control group. Peltoniemi et al. reported a prolongation of 2–2.5 hours in the former group depending on the volume of enrichment used. Tanikawa et al. highlighted a shorter mean surgical time for the control group than the intervention group, with 80 minutes and additional 45 minutes in turn.

#### Number of fat grafting sessions

Five studies reported the number of fat grafting sessions that took place. Three studies (Chang et al 2013, Gentille et al 2015 and Koh et al 2012) used the same number of fat injections for both the SCEFG and AFG groups. The other two studies (Bashir et al 2019 and Sterodimas et al 2011) had an average of 1 session for the SCEFG group and 2 sessions for the AFG group.

#### Subgroup Analysis – Volume Retention

**Fig. 8:**
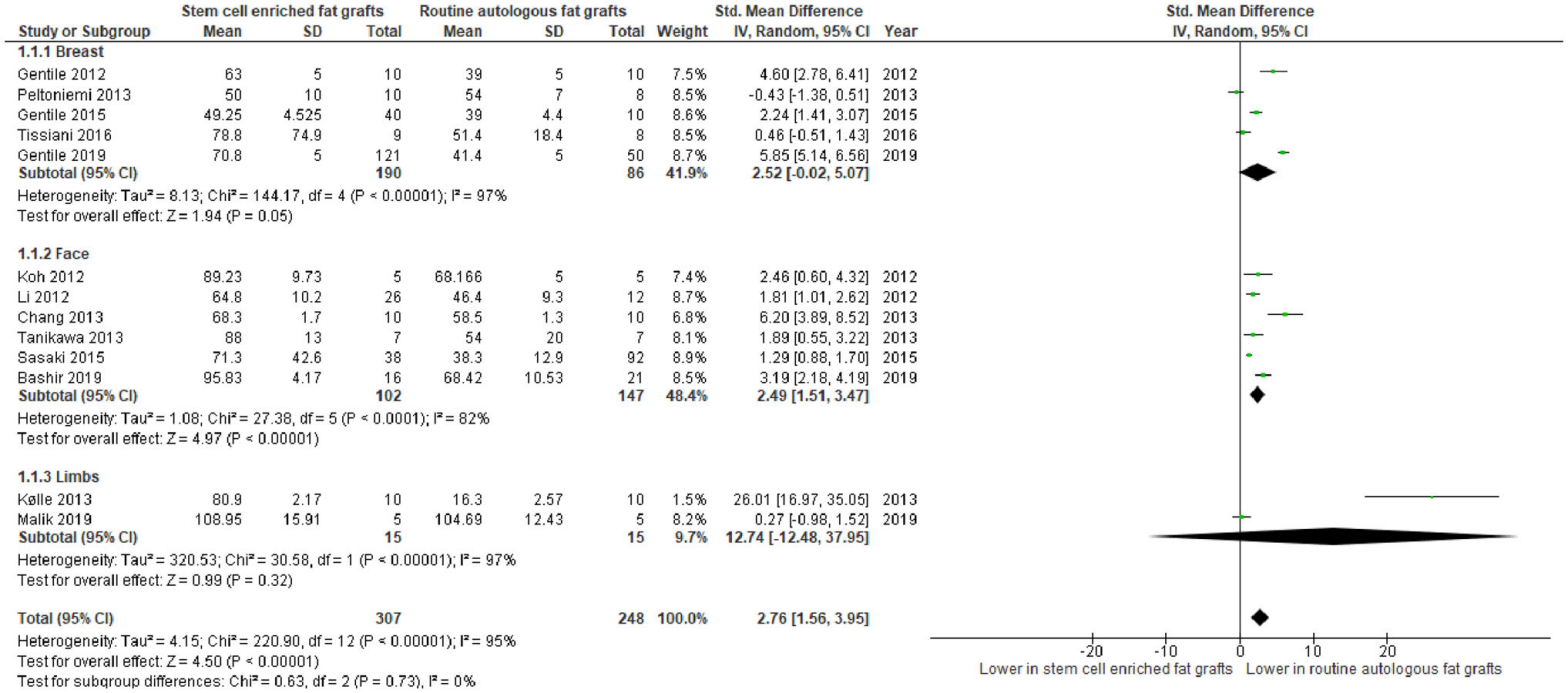
Forest plot depicting subgroup analysis of volume retention for stem-cell enriched fat grafts vs routine autologous fat grafts- Quantitative analysis showing significant differences seen for participants undergoing breast and face grafts and no differences between the subgroups

In Fig. 8, subgroup analysis was done showcasing mean difference in volume retention across 13 different studies with a total number of 555 participants. Overall, there is a significant difference seen in the mean difference analyses favouring the intervention group whereby volume retention is higher (MD:2.76, CI: 1.56–3.95, *P*<0.00001) compared to the AFG group. Additionally, there is no significant difference seen between the subgroups (*P*=0.73).

#### Methodological Quality and Risk of Bias Assessment

The Cochrane Collaboration’s Tool was used to summarise the risk of bias in randomised studies (Table 2). The Newcastle-Ottawa Scale [16] was used to assess the quality of the three non-randomised studies, which offers a star system for analysis (Table 3). Although the comparability was low in most studies, selection and exposure were of higher quality. Overall, all studies were of good quality except for Sasaki et al which was of fair quality based on the AHRQ standards [16].

**Table 2:** Assessment of risk of bias of the randomised trials using the cochrane collaboration’s tool

First author	Bias	Authors’ judgement	Support for judgement
Sterodimas et al. (2011)	Random sequence generation (selection bias)	Unclear risk	No information regarding the randomisation technique is mentioned
	Allocation concealment (selection bias)	High risk	No allocation concealment is done.
	Blinding of participants and personnel (performance bias)	Unclear risk	No blinding of participants mentioned
	Blinding of outcome assessment (detection bias)	Unclear risk	No information given
	Incomplete outcome data (attrition bias)	Low risk	All outcome data reported
	Selective reporting (reporting bias)	Low risk	The study protocol is available and all data is reported in the pre-specified way.
	Other bias	Unclear risk	Similar baseline characteristics not mentioned
Koh et al. (2012)	Random sequence generation (selection bias)	Unclear risk	No information regarding randomisation technique is mentioned
	Allocation concealment (selection bias)	Unclear risk	No information regarding allocation concealment is mentioned.
	Blinding of participants and personnel (performance bias)	Unclear risk	No information given
	Blinding of outcome assessment (detection bias)	Unclear risk	No information given
	Incomplete outcome data (attrition bias)	High risk	Standard deviation for volume differences is missing
	Selective reporting (reporting bias)	Low risk	Study protocol available with no missing outcomes.
	Other bias	Low risk	Similar baseline characteristics
Kølle et al. (2013)	Random sequence generation (selection bias)	Low risk	allocation sequence was generated using an online randomisation generator
	Allocation concealment (selection bias)	Low risk	Allocation was concealed by a person unrelated to the trial management group and monitored by the Good Clinical Practice unit
	Blinding of participants and personnel (performance bias)	Low risk	“The participants, study personnel, and outcome assessors were all blinded to treatment allocation, and blinding was maintained until all data had been analysed”.
	Blinding of outcome assessment (detection bias)	Low risk	“The participants, study personnel, and outcome assessors were all blinded to treatment allocation, and blinding was maintained until all data had been analysed”.
	Incomplete outcome data (attrition bias)	Low risk	All outcome data is reported
	Selective reporting (reporting bias)	Low risk	Study protocol is available in the appendix
	Other bias	Low risk	No further biases detected
Tanikawa et al. (2013)	Random sequence generation (selection bias)	Low risk	Random number generator used
	Allocation concealment (selection bias)	Unclear risk	No information given
	Blinding of participants and personnel (performance bias)	Low risk	Double blinded
	Blinding of outcome assessment (detection bias)	Unclear Risk	No information given
	Incomplete outcome data (attrition bias)	Low risk	No outcome data was missing
	Selective reporting (reporting bias)	Low risk	Study protocol available with no missing outcomes
	Other bias	Low risk	No further biases detected
Malik et al. (2019)	Random sequence generation (selection bias)	Low risk	Patients were selected using envelope method
	Allocation concealment (selection bias)	Unclear risk	No information given regarding concealement
	Blinding of participants and personnel (performance bias)	Low risk	Patients were divided randomly using computerized methods
	Blinding of outcome assessment (detection bias)	Unclear risk	No mention of how outcomes were assessed
	Incomplete outcome data (attrition bias)	Low risk	All outcome data was reported and no patients lost to follow up
	Selective reporting (reporting bias)	Low risk	Study protocol available with no missing outcomes
	Other bias	Unclear risk	No mention of baseline characteristics between the patients

**Table 3:** Newcastle-Ottawa Scale (NOS) to assess the quality of non-randomised studies

Study	Selection	Comparability	Exposure
Yoshimura et al. (2008)	***	*	**
Gentile et al. (2012)	***	*	***
Li et al. (2012)	***	**	***
Chang et al. (2013)	***	*	**
Peltoniemi et al. (2013)	***	*	**
Gentile et al. (2015)	***	*	**
Sasaki (2015)	**	*	***
Tissiani et al. (2016)	***	*	**
Calabrese et al. (2018)	***	**	**
Gentile et al. (2019)	***	**	**
Bashir et al. (2019)	***	*	**

## Discussion

SCEFG produced a superior effect when compared with AFG in terms of mean volume retention shown by the results of the analyses. There was a significant (*P* < 0.0001) improvement in the Intervention group compared with the control groups in the combined analysis(Fig. 2) and the subgroup assessment showing no differences between them (Fig. 8). Conversely, there was a significantly (*P* = 0.003) increased rate of redness and swelling in the experimental group (Fig. 5). However, there were no differences observed in the analyses of fat necrosis (*P* = 0.26) and cancer recurrence (*P* = 0.58) in Fig.s 3 and 4. Similarly, the rates of infection (*P* = 0.48) and cysts (*P* = 7.18) showed no significant differences as demonstrated in Figs. 6, 7. This shows that SCEFG improves mean volume retention whilst not increasing the overall risk of complications. Regarding the between-study heterogeneity, it was low to moderate for cancer recurrence (I^2^ = 0%), cysts (I^2^ = 0%) and fat necrosis (I^2^ = 31%); however, it was considerably high for volume retention (I^2^ = 95%). The heterogeneity assessment was not applicable for the redness and swelling.

In addition to the aforementioned outcomes, the results of the current study reported several secondary outcomes that proved SCEFG to have similar effects to the AFG. In the long-term, there were no significant differences noted between both groups in terms of patient satisfaction post-operatively and scar formation. However, operative time was reported to be longer in the SCEFG group.

SCEFG have been developed to overcome the unreliability of the final volume retention of AFG as well as to reduce complications [35]. The current study findings emphasise that SCEFG is superior to AFG. This is supported by the several proposed mechanisms whereby stem cell enrichment increases adipose survival [12]. Initially stem cells differentiate into adipocytes and endothelial cells assist in adipose tissue regeneration and angiogenesis, respectively [12]. Subsequently, stem cell growth factor release helps to protect surrounding tissues from ischaemia and hypoxia [12, 36]. Finally, they survive as original adipose-derived stem cells [12]. Other studies within the literature have reported similar findings to the results of the current review with Lu et al. concluding that there is an enhancement in the survival and quality of transplanted fat tissues with adipose derived stem cells transduced with vascular endothelial growth factor (VEGF) [37]. A further study by Zhu et al. showed that the supplementation of adipose-derived regenerative cells does not only increase adipose graft retention by 2-fold at both 6 and 9 months post-operatively but also enhances the quality of the grafts with higher capillary density, demonstrating the effect of promoting neovascularisation [38].

Several studies question the safety of stem cell application in oncological cases. Stem cells are suggested by several studies to enhance breast tumour growth [39–42]. Similarly, other studies highlight an increased risk of tumour recurrence with their application [43, 44]. The quantitative assessment conducted by the current meta-analysis however shows no significant difference in the cancer recurrence rate between both stem cell enriched and standard fat transfer cohorts.

While the outcomes of this review and the numerous benefits of SCEFG offer promising results for future practise, there are several limitations that must be considered adapting them in routine clinical practice. ADSC isolation is challenging, with most included studies in this review employing SVF instead [45]. The paucity of published clinical research, a lack of standard methodology, and economic impedance all pose a hinderance to the use of ADSC- or SVF-based cell therapy in clinical activity. Furthermore, despite substantial increases in volume retention when comparing SCEFG for breast and face, none of the included studies for breast reconstruction were prospective or randomised thus reducing the quality of evidence.

Taking into consideration the results from the most reliable evidence and the physiological advantages of stem cells [12, 36], the enhancement of stem cells in AFG should be a routine practice for the management of patients undergoing reconstructive operations. However, in cases of cancer, it is important to be cautious with stem cell enrichment before the establishment of cancer remission [42].

A summary of the best available evidence was obtained by applying a systematic approach to assess the risk of bias of relevant studies [17–32]. The 16 studies were standardised in terms of their design and included patients undergoing reconstructive surgeries. The included studies were homogenous in both the intervention of interest used and the comparative routine grafting, allowing non-biased comparison. However, the current meta-analysis should be reviewed in terms of inherit limitations. 16 studies were included enrolling a total sample size of 686 subjects, only 5 of which were RCTS which could be inadequate to draw definitive conclusions as there is a higher selection bias. Further randomised clinical trials are required to fully assess the efficacy and safety of SCEFG in reconstructive cases.

## Conclusions

The findings of this meta-analysis involving 16 studies comparing stem cell-enriched fat grafts and routine fat grafts suggest that stem cell enrichment improves certain outcomes in patients undergoing reconstruction surgery including the mean volume retention and patient satisfaction and surgical complications, except for redness and swelling. SCEFG remain a promising alternative to AFG bearing in mind issues with cost and lack of standardised protocols. The authors highlight the requirement of further randomised clinical studies to enhance the evidence base for the efficiency of SCEFGs.
